# IL-36RN gene: key insights into its role in pediatric pustular psoriasis pathogenesis and treatment

**DOI:** 10.3389/fped.2025.1520804

**Published:** 2025-03-19

**Authors:** Ye Wang, Mingyue Li, Changcheng Hou, Yueyue Wang, Jing Guo, Xurui Wang

**Affiliations:** ^1^ School of Medicine, University of Electronic Science and Technology of China, Chengdu, China; ^2^ Health Care Department, Hospital of Chengdu University of Traditional Chinese Medicine, Chengdu, China; ^3^ Department of Chinese Medicine Surgery, Jiangsu Province Hospital of Traditional Chinese Medicine Chongqing Hospital, Chongqing, China; ^4^ Dermatological Department, Hospital of Chengdu University of Traditional Chinese Medicine, Chengdu, China; ^5^ Department of Chinese Medicine Surgery, Sichuan Provincial People’s Hospital, University of Electronic Science and Technology of China, Chengdu, China

**Keywords:** IL-36RN, gene mutation, treatment, pediatric, pustular psoriasis

## Abstract

Pediatric pustular psoriasis (PPP) is an autoimmune skin disease that seriously affects the physical and mental health of children. The IL-36RN (Interleukin-36 Receptor Antagonist) gene plays a key role in the pathogenesis of PPP. This review comprehensively elaborates on the research progress of IL-36RN in the context of the pathogenesis and treatment of PPP, covering the basic structure, function, mutation sites and types, and inheritance patterns of the gene and its role in the pathogenesis of PPP. In addition, we discussed the frequency of IL-36RN mutations in patients with different types of PPP and the treatment methods for these patients, aiming to provide a valuable reference for further research and treatment of this disease.

## 1 Introduction

Pustular psoriasis is classified as localized and generalized (GPP). Pediatric pustular psoriasis (PPP) is a more severe subtype observed among children. The incidence of GPP is approximately 0.6%–1.7%, whereas that of PPP is approximately 0.05%–0.16% (1). GPP is potentially life-threatening and is characterized by a sudden onset and recurring attacks of a generalized rash with diffuse pustules, accompanied by fever and biochemical abnormalities such as increased white blood cell counts and serum C-reactive protein (CRP) levels (2). PPP manifests as sterile pustules ranging from a needle tip to millet in size on an erythematous base or normal skin (3). Some pustules merge into pustular lakes, whereas some are distributed on the edges of erythema. In severe cases, erosion, exudation, and pus scabs may appear (4). The skin lesions can be found across the body, mainly on the flexural surfaces of the limbs and in wrinkled areas (5). In addition, skin thickening, deformation, and subungual pyogenic accumulation may be observed. Children with PPP often have systemic symptoms such as itching, fever, mucosal damage, joint swelling and pain, and secondary skin infections (5).

Although the etiological factors and pathogenesis of PPP remain unclear, genetic, environmental, and immune-related factors are considered to be involved (6). Some scholars have found that PPP is related to type 1 endoplasmic reticulum aminopeptidase, HLA-C*06 (The Human Leukocyte Antigen-C*06, formerly HLA-Cw6) positivity, and IL-36RN mutations (7). Moreover, infections (mostly upper respiratory tract infections), drugs, mental stress, and trauma can induce and aggravate the condition (8). The average age of patients with PPP at onset is reported to be 6.3 ± 4.9 years;the younger the age, the more severe the condition. Notably, the incidence rate of PPP is slightly higher in boys than in girls (9). In recent years, several studies have shown that IL-36RN mutations can directly lead to the occurrence of PPP and that the frequency of these mutations in patients with PPP is 78.5% (10). However, these mutations are also found in 10.7% of the general population (10). Although mutations in genes such as Serine Protease inhibitor gene serpin family A member 3 (SERPINA3) (11), Caspase Recruitment Domain-containing protein 14 (CARD14) (12), and Adaptor Protein Complex 1 Subunit sigma-3 (AP1S3) (13) have all been found to be potentially associated with PPP, the mutation in the IL36RN gene is the most common. This review summarizes the research progress of IL-36RN mutations in the context of the onset and treatment of PPP, which is of great significance for improving the understanding of the pathogenesis of this disease and its treatment strategies.

Additionally, there is no clear conclusion regarding the differences in IL-36RN mutation sites between adults and children in terms of this disease. However, some studies have mentioned some common mutation sites specific to adults and children respectively. In patients with generalized pustular psoriasis (GPP), Li Ming (14) found that there was a statistically significant difference in the mutation frequency of the IL36RN gene carried by pediatric generalized pustular psoriasis (PGPP) and adult generalized pustular psoriasis (AGPP) (*P* = 0.008). This indicates that there are differences in the gene mutation rates between adults and children. However, currently, there is no result showing that there are differences in the gene mutation sites between them.

## 2 Structure and function of the IL-36rn gene

### 2.1 Gene location and family affiliation

The IL-36RN gene is located on the long arm of chromosome 2 (2q14.1) and is an important member of the interleukin (IL) family, especially playing a key role in regulating the signaling network of the IL-1 family (15). IL-36RN, along with other related genes, constitutes a complex cytokine regulatory system that participates in maintaining the body's immune balance and regulating inflammatory responses normally (16). IL-36RN has certain similarities and associations with other Interleukin family members in terms of structure and function, and these genes interact with each other to regulate the immune response (17).

### 2.2 Encoded protein and its mechanism of action

The IL-36RN gene encodes interleukin 36 receptor antagonist (IL-36Ra), a protein consisting of 153 amino acids (18). The IL-36 family comprises the inflammatory cytokines IL-36α, IL-36β, and IL-36γ (19). Under physiological conditions, these cytokines bind to the IL-36 receptor, recruit the IL-1 receptor accessory protein, and activate the nuclear factor κB (NF-κB) and mitogen-activated protein kinase signal transduction pathways, thereby strengthening the inflammatory response (20). However, IL-36Ra can bind to IL-1 receptor-related protein 2 and competitively antagonize IL-36,inhibit the pro-inflammatory effects of IL-36α, IL-36β, IL-36γ, and the downstream signaling pathways and prevent the occurrence of an inflammatory response (21). This precise regulatory mechanism ensures that the body can maintain the balance of the inflammatory response under physiological conditions and prevent excessive inflammation from causing damage to tissues (22). Specifically, the binding site of IL-36Ra in IL-1 receptor-related protein 2 is highly specific. A strong binding between these proteins effectively blocks the binding of IL-36 family members to IL-1 receptor-related protein 2 (23). When IL-36Ra binds to IL-1 receptor-related protein 2, it undergoes a conformational change that enhances its antagonistic effect on IL-36 family members (24). In addition, IL-36Ra can regulate intracellular signal transduction molecules, such as inhibition of the activities of some kinases, to indirectly affect the signal transduction of downstream inflammatory pathways, thereby achieving fine regulation of the inflammatory response (25).

## 3 IL-36RN mutations in pediatric pustular psoriasis

### 3.1 Mutation sites and types

So far, 11 IL-36RN mutations (p.Arg10X, p.Leu27Pro, p.Lys35Arg, c.115 + 6T>C, p.Asn47Ser, p.Arg48Trp, p.Pro76Leu, p.Arg102Gln, p.Arg102Trp, p.Ser113Leu, and p.Thr123Arg) have been identified in African (10), European (26), and Asian (27, 28) populations. In the current study, 4 IL-36RN mutations were found in the GPP cohort. The mutation c.115 + 6T>C is the most common mutation in both GPP and normal controls. This mutation has been reported in Japanese and Malay populations, but not found in European and African populations, indicating that the IL-36RN mutations in Chinese populations are distinctly different from those in European and African populations (14).

#### 3.1.1 Common mutations in Asian populations

Asian patients with PPP exhibit some specific sites and types of IL-36RN mutations. Especially c.115 + 6T>c, c.227C>T, and c.115 + 6T>c may be the main IL-36RN gene variations in Asian populations. A total of 10 mutations have been found in Chinese patients with PPP. The most common mutation is c.115 + 6T>C, which is found in approximately 63.6% of children with PPP. The second most common mutation is p.Ro76leu (10.6%), followed by p.Var57ile, p.Ro82leu, p.Asn47Ser, p.Thr123Met, p.Glu112Lys, p.Pro76Leu, and p.Arg102Gln. These mutations may affect the normal structure and function of IL-36Ra and interfere with its inhibitory effect on the inflammatory response (18). The c.115 + 6T>C mutation can lead to exon 3 skipping, causing a frameshift mutation and the premature appearance of a stop codon, which impairs the expression and antagonistic function of IL-36Ra (29). In addition, other mutations, such as p.Thr123Arg, have been found in some Asian patients with PPP. The p.Thr123Arg mutation leads to incorrect folding of IL-36Ra, resulting in an unstable protein structure. *In vitro* studies have shown that p.Thr123Arg impairs the expression of IL-36Ra, preventing it from blocking the signal transduction pathway of IL-36 (30).

#### 3.1.2 Differences in IL-36rn mutations among different races

IL-36RN mutations are more complex and diverse across races. The types of IL-36RN mutations in Asian populations are significantly different from those in European and African populations. The most common IL-36RN mutation in Asian patients with PPP, C.115T>C, has not been found in European or African populations. However, other types of mutations such as missense and nonsense mutations (p.Leu27Pro and c.338 C>T [p.Ser113Leu) have been found in European populations, and other specific mutations have been found in African populations (31). The difference in the distribution of IL-36RN mutations across races suggests the potential influence of genetic background on the development of diseases and may be related to the genetic susceptibility of different races (32).

The IL-36RN mutations found in African patients with PPP have been shown to affect the expression and function of IL-36RN; however, their specific mechanisms of action may be different from those of mutations found in Asian and European patients with PPP. This racial difference may be reflected in clinical manifestations and treatment responses and warrants further in-depth investigation.

### 3.2 Inheritance pattern

PPP caused by IL-36RN mutations is a spontaneous disease and follows an autosomal recessive inheritance pattern (33). Therefore, a child must inherit a mutated allele from each parent to develop the symptoms of PPP. This inheritance pattern determines the transmission law of the disease in a family and is important for genetic counseling and disease prevention. A genetic tendency can be observed in some familial cases (34). If both parents are heterozygous carriers (i.e., carrying one normal allele and one mutated allele), their children have a 25% probability of inheriting two mutated alleles and developing the disease, a 50% probability of becoming heterozygous carriers, and a 25% probability of inheriting two normal alleles and not developing the disease. The calculation of this genetic probability is necessary for predicting the risk of diseases in a family (35).

## 4 Pathogenesis of pediatric pustular psoriasis caused by IL-36rn mutations

### 4.1 Structural change and functional loss in the encoded protein

IL-36RN mutations alter the structure of the encoded protein IL-36Ra (36). This structural change may involve amino acid substitutions, deletions, or insertions, which attenuates the binding of IL-36Ra to IL-1 receptor-related protein 2 and inhibits its antagonistic effects on IL-36. In some mutation types, amino acid substitutions may alter the spatial conformation of IL-36Ra, hindering its binding to IL-1 receptor-related protein 2 and suppressing its antagonistic function (10). A classic example of this type of mutation is p.Leu27Pro, a missense mutation (34). In addition, some mutations may affect the transcription or translation of the IL-36RN gene, resulting in a decrease in the number of synthesized IL-36Ra proteins and a reduction in the expression of the protein (35). This decrease in the amount and expression of IL-36Ra directly affects its inhibitory effect on the inflammatory response, making the response more likely to occur (37).

### 4.2 Inflammatory pathway activation

**Figure 1:**
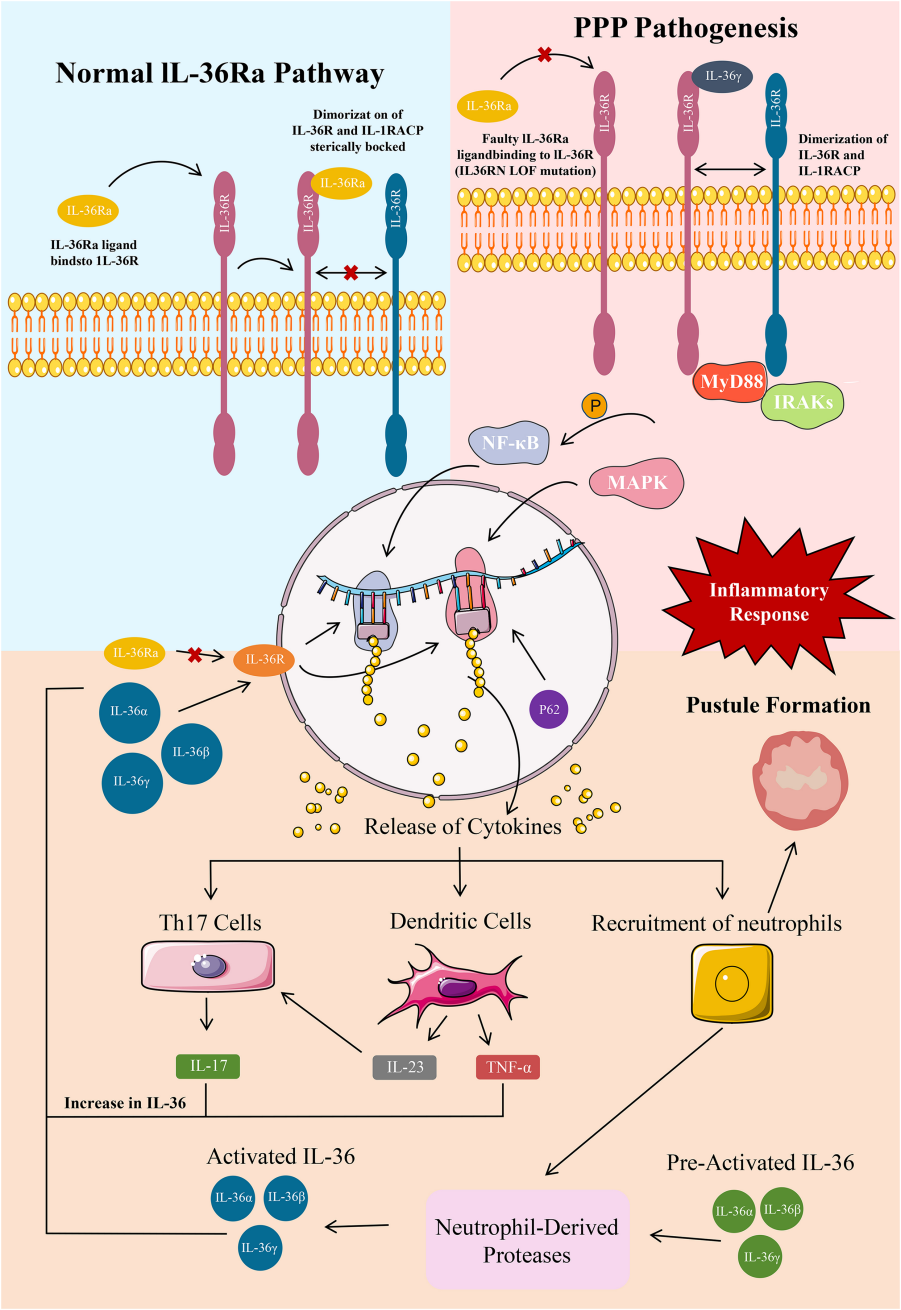
A:PPP Pathogenesis ; B:Epidermal IL-36 pathway in healthy and PPP. A:GPP pathogenesis primarily constitutes a large IL-36-dominated keratinocyte cytokine storm and epidermal neutrophil aggregation. Epidermal neutrophil recruitment leads to pustular skin symptoms and this recruitment is sustained and long-lived due to an MPO deficiency. These keratinocyte cytokines stimulate various inflammatory mediators such as Th17 cells, dendritic cells, and neutrophils. Th17 stimulation causes a release of IL-17A, which increases IL-36 levels. Dendritic cells release TNF-α and IL-23, which further stimulate Th17 and increase IL-36 levels. An MPO deficiency enhances the activity of neutrophil-derived proteases, which continue to activate IL-36.58 An IL-36RN LOF mutation is the primary mutation implicated in GPP that produces dysfunctional IL-36Ra, which normally functions to inhibit IL-36R, stop cytokine production, and prevent inflammation. With no inhibitory protein for IL-36R, large levels of IL-36 constitutively activate IL-36R, which further activates NF-κB and MAPK for more release of proinflammatory cytokines in a constant positive loop. Various other mutations add to this increase in IL-36. An AP1S3 LOF mutation causes P62 buildup leading to NF-κB activation and further IL-36 release. A CARD14 GOF mutation also *ο*veractivates NF-κβ for additional cytokine release. B:IL-36 is mainly expressed in epidermal, bronchial, intestinal epithelial, and various immune cells. The IL-36 pathway regulates the balance of pro-inflammatory and anti-inflammatory cytokine production at these tissue sites. IL-36Ra is an IL-36R antagonist and immune regulator. The Asp148 residue in the β11/12 loop of IL-36Ra prevents heterodimerization of IL-36R and the accessory protein IL-1RAcP and attachment of IL-36 agonists through steric hindrance, blocking an inflammatory response through the pathway.

Owing to the weakened antagonistic function of IL-36Ra, the binding of IL-36α, IL-36β, and IL-36γ to IL-1 receptor-related protein 2 strengthens correspondingly (24). After successful binding, these cytokines activate pro-inflammatory signaling pathways, such as NF-κB and mitogen-activated protein kinase pathways, eventually resulting in skin inflammation (24) (Figure 1).

In patients with PPP, this inflammation mainly manifests as pustules, erythema, and scales on the skin, seriously affecting the skin health and quality of life of the patients (38). Activation of NF-κB leads to upregulation of various inflammation-related genes, such as tumor necrosis factor alpha (TNF-α), interleukin 6 (IL-6), and interleukin 8 (IL-8), which exacerbate skin inflammation (39). In particular, activated NF-κB enters the nucleus and binds to specific DNA sequences, promoting the transcription of inflammation-related genes (40). For example, an increase in the transcription of the TNF-α gene results in the synthesis of a large amount of TNF-α protein, which is an important pro-inflammatory factor. This protein activates inflammatory cells, such as macrophages and neutrophil, and stimulates them to release more inflammatory factors, eventually resulting in the amplification of inflammatory responses (41). In addition, the activation of the mitogen-activated protein kinase pathway can also affect the inflammatory response (42). This pathway regulates the proliferation, differentiation, and apoptosis of cells. Under inflammatory conditions, this pathway can promote the abnormal proliferation and differentiation of keratinocytes, leading to pathological changes in the skin (43). For example, it can induce over-proliferation of keratinocytes, resulting in the formation of thick scales, and affect the normal metabolism of skin cells, causing damage to skin barrier function (44) (Figure 1).

## 5 Prevalence of IL-36rn mutations in patients with different types of pediatric pustular psoriasis

### 5.1 Differences in mutation prevalence between isolated and associated types

Pustular psoriasis is a group of severe skin diseases characterized by repeated eruptions of pustules filled with neutrophils, including acute systemic (GPP) or chronic localized (ACH) manifestations, and can be complicated by psoriasis vulgaris (PV). IL36-RN mutations show significant differences among subtypes of pustular psoriasis. In patients with GPP, IL36RN mutations are relatively common, with a carriage rate of 23.7%, and are more prone to biallelic mutations. In ACH patients, the carriage rate of this mutation is 18.2%. However, in PPP patients, IL36RN mutations are relatively rare, only 5.2% (*P* = 1.9 × 10^−14^ compared with GPP, *P* = 0.0018 compared with ACH). Nevertheless, IL36RN mutations are significantly associated with an earlier onset age in all three subtypes (*P* = 0.003) (38). These differences indicate that IL36RN mutations play different roles in different subtypes of pustular psoriasis, which is of great significance for understanding the pathogenesis, diagnosis, and treatment of the disease. The pathogenesis of clinical subtypes of PPP may be related to specific IL-36RN mutation patterns or the synergistic action of other genes. Some clinical subtypes of PPP may be more sensitive to IL-36RN mutations, whereas the development of other subtypes may require the combined action of IL-36RN and other genes. For example, some genes may have abnormal expression or mutations in some PPP subtypes, and these genes may interact with IL-36RN, collectively affecting the occurrence and development of the disease (45). These differences emphasize the clinical complexity and heterogeneity of PPP, necessitating further investigation into the pathogenesis of clinical subtypes of PPP to provide a basis for precise treatment.

## 6 Treatment methods and progress of the treatment of pediatric pustular psoriasis based on IL-36rn mutations

### 6.1 First-line treatments

Acitretin, a retinoid, is considered a first-line drug for the treatment of PPP (46). Its mechanism of action is quite complex. Acitretin can promote the differentiation of keratinocytes in the normal direction, reduce the number of abnormal keratinocytes, and inhibit the over-proliferation of keratinocytes (47). It has demonstrated a certain degree of treatment efficacy in some patients with PPP in clinical settings. It can relieve symptoms by reducing the number and size of pustules and improving erythema and scales (48). Choo et al. treated a 15-week-old patient with PPP with acitretin at an initial dose of 5 mg/day and subsequently adjusted the dose according to the clinical response, ranging from 1.5 to 7 mg/day. After continuous treatment for 22 months, the oral administration of acitretin was discontinued. Finally, only local treatment, humectants, and oral supplements were found to have good therapeutic effects against PPP (49). The therapeutic effects of acitretin may vary across patients, and some patients may have a poor treatment response. Therefore, developing other treatment methods for PPP is necessary.

Spesolimab is a humanized monoclonal antibody against IL-36 receptor, which has shown good efficacy and safety in adult and pediatric patients with GPP. In a clinical treatment of 5 pediatric patients aged 4.8–10.6 years, after one week of dosing with spesolimab, the total GPPGA score of all patients was 0/1, the pustule subscale was 0, and the GPPASI of all patients was 50. The GPPASI of 4 patients was 75. Meanwhile, plasma IL-36 α The levels of IL-36 β, IL-36 γ, IL-17A, IL-17C, IFN-γ, TNF, IL-6, and IL-8 decreased on average, while the levels of IL-36 α, IL-36 β, IL-17A, IL-17C, and IL-6 were statistically significant. There is no recurrence after 2–8 months of treatment. Except for one patient who experienced upper respiratory tract infection in the first week, no other adverse events were recorded. Therefore, Spesolimab may be a prospective choice for children aged 4–12 (50).

### 6.2 Combination therapy

#### 6.2.1 Combination of immunosuppressants

Owing to the limitations of single-drug treatment, combination therapy has become an important strategy for improving treatment effects. Combining cyclosporin A (CsA) or methotrexate with other immunosuppressants is a common therapeutic strategy for PPP.

CsA can inhibit the activity of calcineurin in T lymphocytes, thereby blocking the activation of T lymphocytes and reducing the release of inflammatory factors, such as TNF-α and IL-6. To evaluate the efficacy and safety of CsA in the treatment of PPP, Baskan et al. conducted a retrospective analysis of 22 patients with PPP aged <18 years who received CsA therapy. The results showed that 17 patients responded well to the treatment. The mean treatment dose was 3.47 ± 0.62 mg/kg/day, the mean treatment duration was 5.68 ± 3.29 months, and the median time for the complete clearance of lesions was 4.0 weeks, indicating that CsA was effective and safe in the treatment of children with psoriasis (51). However, owing to the risk of malignancy and lymphoproliferative diseases, CsA therapy should be limited to a short course (52).

Methotrexate interferes with cellular metabolism and inhibits cell proliferation, thereby suppressing the inflammatory response to a certain extent. It can be administered to patients either orally or via injection. Subcutaneous injection improves the bioavailability of methotrexate and reduces the risk of adverse digestive events (52). Methotrexate can be used to treat pustular psoriasis. In a retrospective study of 157 patients with multiple types of psoriasis (including 12 patients with GPP), 31% of the patients showed clearance of skin symptoms after 3–5 months of treatment with methotrexate (53).

The combined use of acitretin and immunosuppressants can exert a synergistic effect and improve treatment efficacy, especially in patients with more severe PPP. In some clinical studies, the effective treatment rate of combination therapy has been shown to be 60%–80%, which is significantly higher than that of single-drug treatments. Both acitretin and immunosuppressants regulate the inflammatory response and cellular metabolism through different mechanisms, which may be a reason for their synergistic therapeutic effects. For example, acitretin regulates the metabolism of keratinocytes, whereas immunosuppressants inhibit the activity of inflammatory cells, and their combination more effectively controls the inflammatory response and improves the symptoms (54).

#### 6.2.2 Combination of biologics

In recent years, biologics have gradually attracted attention in the treatment of PPP. Biologics are a class of drugs prepared using biotechnology. They have high specificity and can precisely act on targets to effectively treat the disease while reducing the adverse effects on normal tissues. Etanercept and adalimumab are common biologics used to treat PPP. These drugs can specifically bind to inflammatory factors or their receptors to block the transmission of inflammatory signals, thus achieving the purpose of treatment (55). In 2021, five biologics were approved in Europe for the treatment of psoriasis in children. These biologics included two anti-TNF-α drugs, namely, etanercept (Enley, Pfizer, and biosimilars) and adalimumab (Humella, AbbVie, and biosimilars); an anti-interleukin-12/23 drug, namely, ustekinumab (Sedanol, Janssen Pharmaceuticals); and two anti-interleukin-17 drugs, namely, secukinumab (Cogentine, Novartis) and ixekizumab (Tuoz, Eli Lilly). The efficacy of these drugs is usually assessed by calculating the percentage reduction in the Psoriasis Area and Severity Index (PASI) score after 12 of the 16 weeks of treatment.

In addition, there are relevant cases of using IL-36 blockers (such as spesolimab/spevigo) to treat GPP. Furthermore, apremilast can also be used for the treatment of psoriasis. As a TYK2 inhibitor, Deucravacitinib has been shown to be superior to apremilast in the treatment of moderate to severe plaque psoriasis (56).

##### 6.2.2.1 Etanercept

Etanercept is a TNF-α inhibitor. It can specifically bind to TNF-α and prevent it from binding to its receptor. In a pivotal clinical trial evaluating the safety and efficacy of etanercept in 106 pediatric patients with moderate-to-severe plaque psoriasis, 57% of the patients achieved a 75% reduction in PASI scores (PASI 75) (57) after 12 weeks of treatment. Similarly, a long-term open-label extension study evaluating the efficacy of etanercept over 5 years showed that the drug had a good long-term safety profile with no adverse events and sustained efficacy (58). In addition, intermittent etanercept therapy (a 12-week discontinue–retreatment regimen) is reported to be safe and effective, with 80% of patients maintaining or regaining PASI 75 at the end of 12 weeks (59). Etanercept has been used as a control in two clinical trials evaluating the use of an anti-interleukin-17 drug for the treatment of severe plaque psoriasis in children. The results of these trials were similar to those of the pivotal trial of etanercept in that 63% of the patients in these trials achieved PASI 75 with a good safety profile (59, 60). However, there is still a lack of large—scale clinical efficacy observations on the treatment of PPP with Etanercept. Currently, only a few case reports suggest that Etanercept holds a positive prospect in treating PPP. One patient showed clinical response within 4 weeks and had no relapse (61). Another patient controlled the relapse through psoralen and ultraviolet A therapy (PUVA) in addition to Etanercept (62). Two patients experienced adverse reactions, one patient had ecchymosis at the injection site (63), and another patient had candidiasis and MRSA infection (62), all of which were successfully treated. Except for the recurrence of pustular lesions in 2 patients (62, 64) and the lack of control over nail dystrophy (61), all 5 patients achieved complete control.

##### 6.2.2.2 Adalimumab

Adalimumab is a monoclonal antibody targeting the interleukin 6 (IL-6) receptor. It can bind to the IL-6 receptor with high affinity, blocking the binding of IL-6 to its receptor and the subsequent signal transmission. Du analyzed the data of patients with PPP aged 2–13 years treated with subcutaneous injection of adalimumab. The results showed that in the first week after the start of treatment, the skin condition of 7 patients improved significantly, the Physician's Global Assessment (PGA) scores of most patients were significantly decreased, pustular lesions were cleared or nearly cleared in some patients, and systemic or laboratory scores were decreased. These results suggested that adalimumab had significant therapeutic effects against PPP in clinical settings (65). A pivotal clinical trial evaluating the efficacy and safety of adalimumab in 38 children with severe plaque psoriasis showed that 57.9% of the children treated with a standard dose of adalimumab (0.8 mg/kg) achieved PASI 75 after 16 weeks of treatment. On the contrary, the half-standard dose (0.4 mg/kg) was less effective, with only 32% of the children achieving PASI 75 during the study period (52).

Ixekizumab is a human monoclonal antibody that selectively targets interleukin-17A.Two pivotal clinical trials involving 115 children with moderate-to-severe psoriasis and 39 children with severe plaque psoriasis have evaluated the efficacy and safety of ixekizumab. After 12 weeks of treatment, 89% and 84% of these patients achieved PASI 75, respectively, and 78% and 75% of these patients achieved PASI 90, respectively (66). A study on Japanese patients with GPP found that after an initial induction dosing of ixekizumab, at Week 12, 4 out of 7 patients had “resolved,” 2 “improved,” and 1 “worsened.” Two continued 80-mg Q2W treatment to Week 20, with 1 “resolved” and 1 “improved.” Efficacy was evident through GIS, PASI 75 achievement, and improvements in PSSI, BSA, and GPP Severity Index. Patient—reported outcomes also showed positive changes. No notable adverse events occurred, indicating a consistent safety profile, suggesting this dosing regimen may be effective and tolerable for patients with GPP (67). Meanwhile, although some case reports of ixekizumab in the treatment of GPP have been found (68, 69), there is still a lack of direct evidence for the treatment of Palmoplantar Pustulosis (PPP) with ixekizumab.

Another commonly used human monoclonal antibody that selectively targets interleukin-17A is secukinumab. Wei (4, 5) analyzed the data of 46 patients with PPP treated with secukinumab and found that GPPASI scores improved in most patients after 4 weeks of treatment. All patients achieved GPPASI 90 or above after 12 weeks of treatment, and these indices were maintained after 24 and 45 weeks of treatment. These results indicate that secukinumab has a good safety profile with rapid action and long-term efficacy in the treatment of PPP.

IL-36 blocker(spesolimab): Spesolimab is a first-in-class IL-36 monoclonal antibody receptor antagonist, approved for the treatment of acute GPP episodes (Figure 1). Spesolimab is a humanized monoclonal antibody against IL-36 receptor, which has shown good efficacy and safety in adult and pediatric patients with GPP. In a Phase 2 clinical trial evaluating the treatment of GPP episodes with a single dose of intravenous spesolimab on Day 8, 54% of patients receiving spesolimab had a GPP Physician's Global Assessment (GPPGA) pustule score of 0, and 43% had a total GPPGA score of 0 (70). Another Phase 2 clinical trial evaluating subcutaneous injections of spesolimab found that by Week 48, patients in the low-dose, mid-dose, and 10% high-dose spesolimab groups experienced episodes. Compared to placebo, the risk ratios for GPP episode duration in the spesolimab groups were 0.16 (*P* = 0.0005), 0.35 (*P* = 0.0057), and 0.47 (*P* = 0.027) (71), respectively. The infection rates in the treatment group and placebo group were similar, and serious adverse events such as drug reactions (e.g., eosinophilia and systemic symptoms, DRESS), cholelithiasis, and breast cancer occurred in the spesolimab group. Spesolimab is a safe and effective treatment for adult acute GPP episodes. Future clinical trials can determine safety and efficacy compared to other drugs (72).

As for its application in pediatric patients with GPP, a recent study has provided some insights.A recent study by Chen et al. (50). Evaluated the use of spesolimab in treating pediatric generalized pustular psoriasis (GPP) Five children aged 4–12 with GPP, who had not achieved long-term remission with previous treatments, were given a single intravenous dose of spesolimab. After 1 week, all patients had significant improvements in GPPGA total score (0/1), pustulation subscore (0), and GPPASI (50, with four patients reaching 75). The levels of multiple cytokines, including IL-36α, IL-36β, IL-17A, IL-17C, and IL-6, decreased significantly. Body temperature normalized in a median of 4 days, and WBC and CRP levels also decreased. No relapse occurred within 2–8 months, and only one patient had a mild upper respiratory infection in the first week. This study suggests that spesolimab may be a promising option for pediatric GPP, but larger and more comprehensive studies are needed to confirm its efficacy and safety.

At present, more and more biologics such as Deucravacitinib have been incorporated into the treatment range of GPP by various countries. After being used for the treatment of adult GPP, they may be applied in the treatment of PPP in the clinical setting.In a phase 3 trial (POETYK PSO—4, NCT03924427), the efficacy and safety of deucravacitinib, an oral selective allosteric tyrosine kinase 2 inhibitor, were evaluated in Japanese patients with moderate to severe plaque psoriasis (PP), generalized pustular psoriasis (GPP), and erythrodermic psoriasis (EP). Seventy—four patients were treated (PP: *n* = 63; GPP: *n* = 3; EP: *n* = 8). At week 16, in the PP group, 76.2% achieved PASI 75 and 82.5% achieved sPGA 0/1, with improvements seen as early as week 4 and responses maintained through week 52. For patients with GPP, PASI 75, PASI 90, PASI 100, sPGA 0/1, and sPGA 0 rates were improved at week 16 and generally maintained to week 52. The most common AEs in PP patients were nasopharyngitis and acne. SAEs occurred at a low rate and did not affect any particular organ system. Overall, deucravacitinib was effective and well tolerated in Japanese PP and a limited number of patients with GPP, although the small sample size for patients with GPP limits conclusive statements about its full effect in this subtype (73).

## 7 Conclusion

The IL-36RN gene plays an indispensable role in the pathogenesis of PPP. Its mutation leads to the loss of gene function, triggering an inflammatory reaction, which results in the development of PPP. The sites and types of IL-36RN mutations vary among patients with PPP of different races, and the proportion of patients with these mutations also varies based on the type of PPP (56). In terms of treatment, acitretin is a first-line drug for PPP and combination therapy and treatment monitoring are important for improving outcomes. In the future, studies should investigate the mechanisms underlying IL-36RN mutations; explore more effective treatment methods, such as novel drugs targeting the IL-36RN gene and its related signaling pathways; and assess the application potential of gene therapy in PPP. In addition, it is necessary to strengthen clinical research on PPP, improve the understanding and diagnosis of different clinical subtypes of PPP, and provide better support for improving the prognosis of children with pustular psoriasis.
